# Particle Size-Fraction Analysis: Gilmour et al. Respond

**DOI:** 10.1289/ehp.1002354R

**Published:** 2010-09

**Authors:** M. Ian Gilmour, Seung-Hyun Cho, Haiyan Tong, John K. McGee, Q. Todd Krantz, Richard W. Baldauf

**Affiliations:** National Health and Environmental Effects Research Laboratory, U.S. Environmental Protection Agency, Research Triangle Park, North Carolina, E-mail: gilmour.ian@epa.gov; National Risk Management Research Laboratory, U.S. Environmental Protection Agency, Research Triangle Park, North Carolina

We thank Müller et al. for their interest in our article (Cho et al. 2009) and concur that the secondary sizing of particulate samples initially collected with a size-selective cascade impactor and then resuspended in solution is useful information. In response to their comments, we prepared additional samples of the particulate matter (PM) in the same manner as in the toxicology study and used dynamic light scattering (Malvern Zetasizer, Model Zen 3600; Malvern Instruments Ltd, Malvern Worcestershire, UK) to determine particle size. We found that the coarse PM near road sample (collected approximately 20 m from the nearest lane of a highway) had an average diameter of 3.4 μm; the fine particles, 1.5 μm; and the ultrafine particles, 0.46 μm. These results were confirmed by electron microscopy of similarly collected samples (Devlin R, personal communication). Because the cut points for the sampler were 2.5–10, 0.1–2.5, and < 0.1 μm, for the coarse, fine and ultrafine particles, respectively, it appears that the ultrafine particles did indeed coagulate to a certain degree, although they remained in a much smaller size range than the other two fractions. Despite the ≥ 4‐fold aggregation, the ultrafine particles clearly had a more prominent effect on the cardiovascular system, whereas the larger particles affected the lung. Further experimentation is required to determine if this is due solely to the particle size or to the chemistry of the material. In addition to clarifying the size of the resuspended particles, we also calculated the solubility and found that coarse, fine, and ultrafine particles from the near road sample were 35, 81, and 85% water soluble, respectively. We believe that the size, chemistry, and solubility can affect the toxicological outcome from particle exposure and agree that these features should be reported wherever practicable.
